# Phylogeography of the Mekong mud snake (*Enhydris subtaeniata*): the biogeographic importance of dynamic river drainages and fluctuating sea levels for semiaquatic taxa in Indochina

**DOI:** 10.1002/ece3.29

**Published:** 2011-11

**Authors:** Vimoksalehi Lukoschek, Jennifer L Osterhage, Daryl R Karns, John C Murphy, Harold K Voris

**Affiliations:** 1 ARC Centre of Excellence for Coral Reef Studies, James Cook UniversityTownsville, Queensland, Australia; 2 Biology Department and Rivers Institute, Hanover CollegeHanover, Indiana; 3 Department of Zoology, Field Museum of Natural HistoryChicago, Illinois; 4 Current affiliation: Department of Biology, University of KentuckyLexington, Kentucky

**Keywords:** Freshwater snake, Homalopsidae, Khorat basin, Mekong River, Pleistocene, Sea levels, Sundaland

## Abstract

During the Cenozoic, Southeast Asia was profoundly affected by plate tectonic events, dynamic river systems, fluctuating sea levels, shifting coastlines, and climatic variation, which have influenced the ecological and evolutionary trajectories of the Southeast Asian flora and fauna. We examined the role of these paleogeographic factors on shaping phylogeographic patterns focusing on a species of semiaquatic snake, *Enhydris subtaeniata* (Serpentes: Homalopsidae) using sequence data from three mitochondrial fragments (cytochrome *b*, ND4, and ATPase—2785 bp). We sampled *E. subtaeniata* from seven locations in three river drainage basins that encompassed most of this species’ range. Genetic diversities were typically low within locations but high across locations. Moreover, each location had a unique suite of haplotypes not shared among locations, and pairwise φ_ST_ values (0.713–0.998) were highly significant between all location pairs. Relationships among phylogroups were well resolved and analysis of molecular variance (AMOVA) revealed strong geographical partitioning of genetic variance among the three river drainage basins surveyed. The genetic differences observed among the populations of *E. subtaeniata* were likely shaped by the Quaternary landscapes of Indochina and the Sunda Shelf. Historically, the middle and lower Mekong consisted of strongly dissected river valleys separated by low mountain ranges and much of the Sunda Shelf consisted of lowland river valleys that served to connect faunas associated with major regional rivers. It is thus likely that the contemporary genetic patterns observed among populations of *E. subtaeniata* are the result of their histories in a complex terrain that created abundant opportunities for genetic isolation and divergence yet also provided lowland connections across now drowned river valleys.

## Introduction

Despite being only 4% of the Earth's land surface, Southeast Asia is a global biodiversity hotspot with 20–25% of the planet's animal and plant species (Woodruff 2010). During the Cenozoic this region was profoundly affected by plate tectonic events, dynamic river systems, changing sea levels and coastlines, and climatic variation, which strongly influenced the ecological and evolutionary trajectories of the Southeast Asian flora and fauna (Fontaine and Workman 1978; Rainboth 1996; Hall and Holloway 1998; Voris 2000; Woodruff 2010). These complex physical, geographic, and climatic processes produced diverse opportunities for dispersal and vicariant events resulting in the outstanding terrestrial and aquatic biodiversity of the region.

Previous studies have identified various geographic features of the region that have likely influenced geographic variation in the distribution of terrestrial, freshwater, and marine species of this region, as well as the patterns of genetic diversity and population structure. In particular, Indochina has a complex geological history, with a number of potentially important physiographic events occurring in geologically recent times (Quaternary) that certainly affected many taxa (see reviews in Rainboth 1996; Inger and Voris 2001; Glaubrecht and Köhler 2004; Woodruff 2010; Ding et al. 2011).

**Figure 1:**
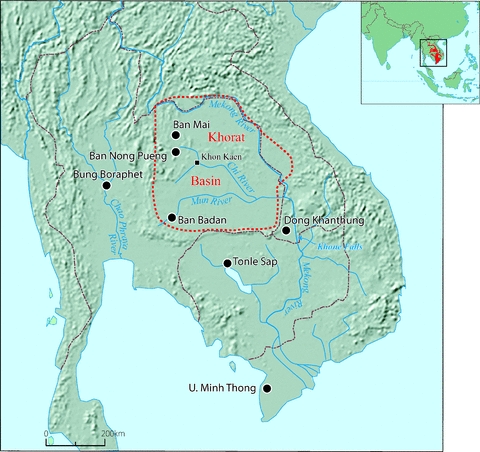
Map showing the seven *E. subtaeniata* collection locations in Indochina. Bung Boraphet is in the Chao Phraya basin, located in the Central Plain of Thailand. Adjacent to the Middle Mekong and in the Khorat Basin (dotted red line) are the geographically proximate Ban Mai and Ban Nong Pueng (collectively referred to as Khon Kaen) in the Chi River catchment and Ban Badan in the Mun River catchment (both tributaries of the Middle Mekong). Dong Khanthung is in Laos above Khone Falls on the southern Middle Mekong. Tonle Sap and U. Minh Thong are in the Lower Mekong (See Appendix A1 for GPS coordinates and other details of sampling locations).

One prominent and ancient geographic feature in Indochina is the Khorat Basin (Fig. 1), which covers an area of 180,000 km^2^ in northeast Thailand (Hutchison 1989; Carter and Bristow 2003). This sedimentary basin is of Mesozoic age and its current plateau-like morphology is the result of erosion following Quaternary tectonic uplifting and tilting along its southern and western margins (Hutchison 1989; Rainboth 1996; Attwood and Johnston 2001; Glaubrecht and Köhler 2004), which created a low mountainous rim (currently 200–1100 m a.s.l.) along its southern and western borders. The Mun and Chi Rivers in the Khorat Basin, which today flow eastward into the Mekong River (Fig. 1), may have been connected to the Chao Phraya River (west) prior to the middle Pleistocene (Rainboth 1996; Glaubrecht and Köhler 2004). There were also major river capture events associated with the Quaternary tectonic uplifting and tilting of the Khorat Basin. For example, historically, the Mekong certainly flowed south to the Gulf of Thailand through what is now the Chao Phraya river plain (Carbonnel 1965; Workman 1977) contributing to the massive Siam River (Rainboth 1996). The contemporary Mekong did not develop until the late Pleistocene (perhaps 10,000 years ago) and assumed its present course from Tibet to Vietnam during this period (Fontaine and Workman 1978; Rainboth 1996). In addition, during the Quaternary, the Great Lake Basin of Cambodia (directly south of the Khorat Basin) was formed by subsidence to the southeast (Fontaine and Workman 1978).

Another prominent historical event is the occurrence of extensive lava flows in locations along the southeastern edge of the Khorat Basin (Carbonnel and Poupeau 1969; Fontaine and Workman 1978) dated to the middle Pleistocene (700,000 years BP) and extending to as recent as 5,000 years BP (Carbonnel 1967). The largest flow created the Bolovens Plateau in southern Laos near the mouth of the Mun River. Later flows resulted in the development of the Khone Falls at the southeast corner of the Khorat Basin, where the Mekong River drops 21 m over a 10-km stretch of rocky rapids and water falls. These lava flows may have periodically prevented the Mun and Chi tributaries from joining the Mekong, and the Khone Falls continues to be a barrier to the movements of some fish taxa (Rainboth 1996; Adamson et al. 2009).

Historical sea-level fluctuations and associated changes in paleo-river drainages have also profoundly affected the distributions and dispersal opportunities of freshwater species. Massive sea-level declines during the Pleistocene repeatedly exposed the Sunda continental shelf, vastly expanding river systems across the emergent Sundaland. These extended paleo-drainages would have created movement corridors for freshwater species across what is today the Gulf of Thailand via river basins, wetlands, and lakes (Voris 2000; Bird et al. 2005; Sathiamurthy and Voris 2006; Hanebuth et al. 2011). By contrast, starting 8,000 years ago and extending to the Holocene Highstand 5,000 years ago, a marine transgression inundated many of the river deltas of this region, including the Chao Phraya (Somboon and Thiramongkol 1992) and Mekong River deltas (Tamura et al. 2009). This inundation displaced the Mekong River delta and its freshwater and brackish habitats more than 200-km inland from today's delta (Tamura et al. 2009). These cyclic sea-level fluctuations resulted in repeated range expansion and contraction of terrestrial and freshwater species (Woodruff 2010; Hanebuth et al. 2011).

Various studies have examined the effects of this complex biogeographical history of Indochina on the community composition, genetic diversity, and population structure of freshwater fishes and invertebrates. For example, similarities in fish community composition between the Chao Phraya and Middle Mekong support the geological evidence for the capture and diversion of the Mekong by the rivers of the Chao Phraya basin (Taki 1975; Rainboth 1996; Yap 2002). Studies using molecular data show a diversity of results, but all support the importance of historical changes in river basins. For example, Hara et al. (1998) found strong genetic differentiation of snakehead fish, *Channa striatus*, between the Chao Phraya River basin and the Mun and Chi tributaries of the Middle Mekong; they also report genetic similarities between the Upper–Middle Mekong (Chiang Rai) and the Chao Phraya populations, indicative of the historical connection between these two river systems. Magtoon et al. (1992) found population level karyotype polymorphisms in the ricefish, *Oryzias minutillus*, that correlated with river systems in Thailand and provide evidence of past historical connections between the Chao Phraya and Mekong systems. Adamson et al. (2009) identified four genetically different stocks of the cyprinid, *Henicorhynchus siamensis*, from the Middle and Lower Mekong and suggest that the Khone Falls (Fig. 1) may be an important barrier. They also report genetic similarity between the Khlong River (west of the Chao Phraya) and one of their Middle Mekong stocks, and suggest the Chao Phraya has acted as a “stepping-stone” between basins. Hurwood et al. (2008) found genetic differentiation among stocks of the cyprinid, *H. lobatus*, from the Chao Phraya and the Middle and Lower Mekong; they report high levels of genetic divergence between the Mun and the Mekong, despite the physical proximity and modern day connection between these rivers. Prasankok et al. (2008) found high levels of differentiation between snail populations from the northern Chao Phraya, the Khorat Basin, and southeast Thailand.

Given the variable results from previous studies and the importance of better understanding the effects of biogeographical features for species and genetic biodiversity in Southeast Asia, we have investigated the effects of geographical features on evolutionary and ecological processes in this region (Alfaro et al. 2004, 2008; Karns et al. 2010a, b) focusing on homalopsid snakes, which dominate the semiaquatic snake communities of Southeast Asia (Karns et al. 2005, 2010a; Murphy 2007). The Homalopsidae (Oriental-Rear-fanged water snakes—37 species currently recognized) are opisthoglyphous (rear-fanged), mildly venomous (Fry et al. 2006), and distributed from Pakistan and the Indian subcontinent across Southeast Asia to northern Australia (Murphy 2007). All homalopsids are semiaquatic, primarily nocturnal, and usually associated with mud substrates. Semiaquatic taxa are particularly interesting with respect to gene flow, dispersal, and speciation processes because of their intermediate ecological position across the continuum of terrestrial–aquatic habitats. This study focuses on *Enhydris subtaeniata*, the Mekong mud snake, a medium sized homalopsid snake found in a variety of freshwater habitats with mud substrates, including rice paddies, streams, ponds, ditches, and canals (Karns et al. 2005, 2010a). It occurs primarily in the Middle and Lower Mekong river basins from Laos, Thailand, Vietnam, and Cambodia; however, one population is known from Bung Boraphet in the central plain of Thailand (Karns et al. 2010a).

This study aimed to improve our understanding of the effects of historical biogeography in Indochina by examining phylogeographic patterns for *E. subtaeniata* using sequences (∼2800 bp) from three mitochondrial genes. We sampled across the three major river drainages that encompass most of this species’ current range and used phylogenetic and population genetic approaches to evaluate several hypotheses about the effects of Quaternary geological and climatic processes on diversification. Population genetic analyses were used to assess the amount of genetic divergence and population genetic structure and we applied known mutation rates to estimate the timing of lineage divergences, and compared these with the timing of geological and climatic events in Indochina during the Quaternary. We then used phylogenetic methods to evaluate the relationships among major evolutionary lineages and the degree of concordance between phylogroups and the geographical configuration of river drainage basins. Finally, isolation-by-distance analyses were used to evaluate whether genetic divergences reflected geographical distances among populations. Our findings indicate that the historical biogeography of Indochina has strongly influence contemporary population structure and these findings are evaluated in the context of previous molecular studies in the region.

## Methods

### Study species and sampling sites

We sampled *E. subtaeniata* (*n* = 48) from three river basins encompassing most of this species’ range (Fig. 1). We defined the Middle Mekong as the Mekong and its tributaries (Chi and Mun) in the northern Khorat Basin to the Khone Falls in the south (*n* = 26); the Lower Mekong as the Mekong and tributaries below the Khone Falls to the South China sea (*n* = 7); and the Chao Phraya in Thailand (*n* = 15). We collected snakes from seven locations in these three river basins: Ban Mai and Ban Nong Pueng (Khon Kaen region) from the Chi tributary of the Middle Mekong; Ban Badan from the Mun tributary of the Middle Mekong, and Dong Khanthung from the Mekong proper; U. Minh Thong and Tonle Sap in the Lower Mekong, and Bung Boraphet in the Chao Phraya (Fig. 1; see Appendix A1 for information on collection sites). Snakes were obtained during Field Museum of Natural History expeditions conducted in 2003, 2004, and 2007 (Karns et al. 2005, 2010a), with additional snakes collected by Bryan Stuart (Stuart et al. 2000; Stuart 2004). Snakes were typically collected as incidental by-catch from local fishers. Live snakes were euthanized, processed, and preserved with formalin. Tissue samples (liver and heart) for genetic analysis were taken from euthanized snakes and preserved in 20% EDTA-salt saturated storage buffer or 95% ethanol. Preserved snakes were deposited in the National Science Museum of Thailand.

### DNA extraction, PCR amplification and sequencing

Total genomic DNA was extracted from liver or muscle tissues using PureGene Animal Tissue DNA Isolation Protocol (Gentra Systems, Inc., Minneapolis, MN). Three mitochondrial fragments were selected for analyses based on their previous usefulness for evaluating phylogenetic and phylogeographic patterns in snakes (Lukoschek and Keogh 2006; Lukoschek et al. 2007; Karns et al. 2010a): ATPase ∼880 bp; ND4 ∼860 bp plus adjacent 3′ tRNA-His and tRNA-Ser ∼80 bp; and cytochrome *b*∼1145 bp. Mitochondrial fragments were amplified and sequenced using the primers (Appendix A2) and protocols described in Karns et al. (2010a). Sequence data were edited in Sequencher (Gene Codes Corporation, Ann Arbor, MI), aligned with Clustal V (default parameters) (Thompson and Gibson 1997), and visually refined. Following alignment, all coding region sequences were translated into amino acid sequences in MacClade v.4.06 (Sinauer Inc., Sunderland, MA) using the vertebrate mitochondrial genetic code. No premature stop codons were observed, thus affording confidence that mtDNA sequences were not nuclear pseudogenes.

### Genetic diversity, F statistics, and population structure

DNA polymorphism was summarized using haplotype and nucleotide diversity statistics (Nei 1987) for the five locations with more than five individuals sampled (Table 1), and across all locations. Pairwise *F*_ST_ and φ_ST_ values were calculated for the ten possible comparisons among these five locations: φ_ST_ using the Tamura–Nei model of sequence evolution. Two Analyses of Molecular Variance (AMOVAs) were performed. The first AMOVA included the five locations with sample sizes >5, in order to evaluate genetic divergence among sampled locations. The second hierarchical AMOVA included all seven sampled locations grouped into three drainage basins, in order to evaluate our biogeographic hypotheses (Appendix A1; Fig. 1). Population genetic analyses were conducted using the computer program ARLEQUIN 3.01 (Excoffier et al. 2005). The significance of variance components and F and φ statistics was tested using 10,000 random permutations and *P*-values were adjusted with sequential Bonferroni corrections for multiple comparisons (Rice 1989).

**Table 1:** Summary population statistics for *E. subtaeniata*: sample size (*n*), number of haplotypes (*N*), haplotype (*h* ± SE), and nucleotide (π ± SE) diversities

Sampling localities	*n*	*N*	*h* ± SE	π ± SE (%)
Ban Nong Pueng, Thailand	9	6	0.92 ± 0.07	0.24 ± 0.14
Ban Mai, Thailand	9	3	0.42 ± 0.19	0.02 ± 0.02
Ban Badan, Thailand	7	1	n/a	n/a
Dong Khanthung, Laos	1	1	n/a	n/a
Tonle Sap, Cambodia	2	1	n/a	n/a
U. Minh Thong, Vietnam	5	4	0.90 ± 0.16	0.07 ± 0.06
Bung Boraphet, Thailand	15	2	0.13 ± 0.11	0.01 ± 0.01
Totals	48	18	0.88 ± 0.03	1.22 ± 0.60

### Gene genealogies and genetic divergence

Gene genealogies were estimated using statistical parsimony (SP) implemented in TCS 1.13 (Clement et al. 2000), and maximum parsimony (MP) and maximum likelihood (ML) conducted in PAUP* (Swofford 2000). SP analyses included all sampled *E. subtaeniata* individuals and the geographical locations of sampled haplotypes were mapped on to the resulting network. SP analyses were run with the maximum connection limit ignored forcing all haplotypes into a single network. MP analyses were conducted using all sampled haplotypes thereby producing unrooted trees and also including outgroup species to root trees. Five *Enhydris* species closely related to *E. subtaeniata* (Alfaro et al. 2008; Karns et al. 2010a) for which sequences of all three mitochondrial fragments were available were used as outgroups. ML analyses were conducted using GTR + I + G model of evolution, determined as the best-fit model by the Akaike Information Criterion in Modeltest (Posada and Crandall 1998). Both MP analyses were performed using heuristic searches with 1000 random stepwise sequence addition replicates and tree-bisection-reconnection (TBR) branch swapping with all sites weighted equally. The ML analysis was performed using heuristic searches with 10 random stepwise sequence addition replicates and TBR branch swapping. Sequence divergences among phylogroups were estimated using uncorrected “*p*” distances and corrected for within location sequence divergences following Avise and Walker (1998).

Brandley et al. (2010) estimated mean evolutionary rates of 0.011 and 0.0079 substitutions site^–1^ million^–1^ years (95% highest posterior densities [HPD] 0.0059–0.013) for mitochondrial cytochrome *b* and ND1 genes, respectively, for a semiaquatic snake. These estimated evolutionary rates correspond to 1.6–2.2% sequence divergences between a pair of lineages per million years (95% HPD 1.2–2.6%), typical of the mitochondrial molecular clock (ML_clock_) (Brown 1985). We tested the ML_clock_ hypothesis using a likelihood ratio test that compared the likelihood scores of the unconstrained best ML tree (ML_best_) and an alternative tree with an enforced ML_clock_ that was constructed in PAUP* (Swofford 2000). The likelihood ratio test (calculated as 2[ln ML_clock_– ML_best_] and tested against a chi-square distribution with df = number of taxa minus two) did not reject the clock hypothesis (χ^2^_df23_ = 26.97, *P* > 0.01). As such, we used the ML_clock_ estimates of Brandley et al. (2010) to evaluate a temporal framework for key divergences among *E. subtaeniata* phylogroups.

### Isolation by distance

Mantel tests of correlations between genetic and geographic distance matrices, implemented in the computer program Isolation by Distance Web Service (IBDWS) (Bohonak 2002: Jensen et al. 2005), were used to test for significant relationships between genetic and geographic distance matrices. We used two sets of geographic distance matrices. The first comprised the shortest straight-line distances between pairs of locations (Fig. 1). However, as *E. subtaeniata* tends to be confined to aquatic habitats, we also tested whether distances through suitable habitats (contemporary drainage basins) correlated better with genetic divergence. Rousset (1997) recommended using φ_ST_/1 –φ_ST_ for Mantel tests, however φ_ST_ values can be unreliable for small sample sizes. As such, we used both φ_ST_/1 –φ_ST_ and corrected “*p*” distances as genetic distances for the Mantel tests.

## Results

### Genetic diversity, F statistics, and population structure

The final alignment comprised 2785 bp (ATPase—810 bp; ND4—696 bp; tRNAs—157 bp; cytochrome *b*—1122 bp) with 123 variable sites (110 transitions, 12 transversions, and one indel) that described 18 putative haplotypes among 48 individuals (Table 1). Haplotype sequences were deposited in GenBank (see Appendix A3 for GenBank accession numbers) and the final alignment can be obtained from Vimoksalehi Lukoschek on request. Overall nucleotide diversity across locations was high (%π = 1.22 ± 0.60 SE), but very low within each location (%π = 0–0.07) except Ban Nong Pueng, for which it was slightly higher (%π = 0.24). Overall haplotype diversity also was high (*h* = 0.88) but within-location haplotype diversities were generally low (0–0.42) (Table 1). The two exceptions were Ban Nong Pueng and U. Minh Thong with haplotype diversities of 0.92 and 0.90, respectively (Table 1). Each sampled location had a unique suite of haplotypes, that is, no shared haplotypes between locations. This haplotype distribution was mirrored in the magnitude of pairwise *F*_ST_ and φ_ST_ values, which were large (*F*_ST_ 0.154–0.909; φ_ST_ 0.713–0.998) and highly significant between all location pairs (Table 2). Strong genetic divergence among locations was further demonstrated by AMOVA, which partitioned 58.9% of the genetic variation among the five locations (with *n*≥ 5) when considering haplotype frequencies alone (*F*_ST_ = 0.589, *P* < 0.001), while 95.9% of the genetic variation was attributed to differences among the five locations when sequence divergences among haplotypes were taken into account (φ_ST_ = 0.959, *P* < 0.001). Hierarchical AMOVA accounting for sequence divergences among haplotypes revealed strong (and statistically highly significant) population subdivision at all levels, with 48.9% of genetic variation partitioned among the three drainage basins (φ_CT_ = 0.489), and 47.5% of genetic variation partitioned among locations within regions (φ_SC_ = 0.929), with an overall φ_ST_ of 0.964.

**Table 2:** Pairwise ØST (below shaded diagonal) values for 10 comparisons between five populations with *n* ≥ 5. ØST values were calculated using the Tamura–Nei model of substitution and significance was tested after 10, 000 permutations. All comparisons were highly significant (*P* < 0.001). Sequence divergences among locations (above shaded diagonal) estimated using the Tamura–Nei model of sequence evolution and corrected for within-location diversity (on shaded diagonal) using the equation (pXY – (pX+pY)/2), where pXY is the sequence divergence between locations X and Y, and pX and pY are the within location sequence divergences of locations X and Y, respectively (Avise and Walker 1998)

Bung Boraphet	U. Minh Tonle Sap	Dong Thong	Ban Nong Khanthung	Ban Badan	Ban Mai	Pueng	
Bung Boraphet	0.000	0.008	0.008	0.021	0.019	0.020	0.019
Tonle Sap	n/a	0.000	0.008	0.022	0.017	0.018	0.018
U. Minh Thong	**0.977**	n/a	0.001	0.020	0.016	0.017	0.017
Dong Khanthung	n/a	n/a	n/a	0.000	0.015	0.014	0.014
Ban Badan	**0.998**	n/a	**0.981**	n/a	0.000	0.011	0.010
Ban Mai	**0.995**	n/a	**0.979**	n/a	**0.991**	0.001	0.003
Ban Nong Pueng	**0.953**	n/a	**0.895**	n/a	**0.875**	**0.713**	0.002

### Gene genealogies and genetic divergence

**Figure 2:**
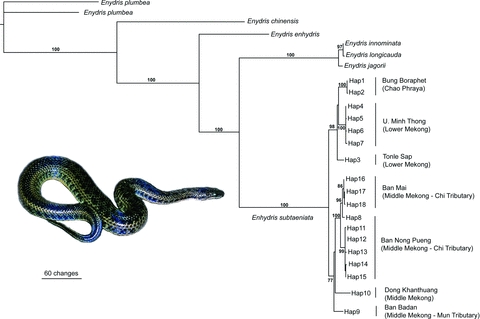
Maximum parsimony (MP) tree showing sampling locations of *E. subtaeniata* haplotypes. Bootstrap support is shown for clades with >70% bootstrap values and four outgroup species: *E. enhydris, E. jagorii, E. innominata*, and *E. longicauda*. The snake photograph taken by JCM is of an *E. subtaeniata* collected at Ban Badan, Thailand in June 2004.

**Figure 3:**
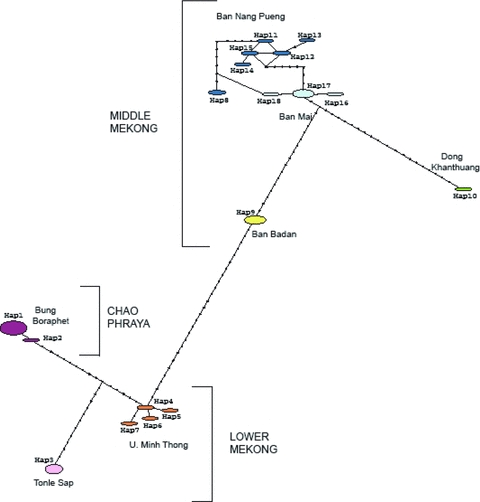
Statistical parsimony (SP) network showing relationships among haplotypes sampled from seven locations from three river drainages: Chao Phraya, Middle Mekong, and Lower Mekong. The SP network was constructed in TCS 1.13 (Clement et al. 2000) with the maximum connection limit ignored forcing all haplotypes into a single network. Unobserved intermediate haplotypes are represented by small filled circles.

MP and ML analyses produced virtually identical rooted trees (Fig. 2); the only differences were the placements of two short internal branches that had no MP bootstrap support. As such, we focus on the MP results. Monophyly of *E. subtaeniata* was strongly supported by parsimony bootstrap values and many intraspecific clades also were well resolved (Fig. 2). Specifically, the four locations with more than one sampled haplotype (Bung Boraphet, Ban Mai, Ban Nong Pueng, U. Minh Thong) each formed a monophyletic phylogroup with >95% bootstrap support (Fig. 2). Haplotypes from the Lower Mekong and the Chao Phraya formed a geographically disjunct monophyletic phylogroup with 98% bootstrap support. Haplotypes from Ban Mai and Ban Nong Pueng from the Middle Mekong (Chi River subbasin) also formed a monophyletic phylogroup with 100% bootstrap support (Fig. 2). Haplotypes from Dong Kanthuang (Laos) and Ban Badan (Mun River subbasin), clustered with the other Middle Mekong haplotypes; however, these affiliations were less well supported (Fig. 2). The SP haplotype network and unrooted MP trees demonstrated congruent relationships among haplotypes that were consistent with the rooted MP tree so only the haplotype network is shown (Fig. 3). The haplotype network highlights the high haplotypic diversities of Ban Nong Pueng, Ban Mai, and U. Minh Thong compared with the other sampled locations and also shows the many unobserved intermediate haplotypes (Fig. 3: “missing” haplotypes are indicated by the small filled circles on the haplotype network).

Percent sequence divergences between U. Minh Thong, Tonle Sap, and Bung Boraphet (Lower Mekong + Chao Phraya phylogroup) ranged from 0.80 to 0.85 (Table 2), suggesting that these populations diverged approximately 350,000–700,000 years ago (based on rate estimates in Brandley et al. 2010). By contrast, sequence divergences ranged from 0.99 to 1.51% among locations in the Middle Mekong (Table 2), the only exception being the low sequence divergence (0.33%) between the geographically proximate Ban Nong Pueng and Ban Mai populations (Fig. 1). Based on Brandley et al.'s (2010) estimated mean evolutionary nucleotide substitution rates, these genetic distances indicate that populations on the Mun, Chi, and Mekong Rivers have been isolated for at least 400,000 years, and possibly as long as 1.8 million years. Finally, sequence divergence between locations in the three drainage basins ranged from 1.63 to 2.23% (Table 2), suggesting that these populations in the Middle Mekong have been isolated from the Lower Mekong and Chao Phraya populations for at least 1 million years and possibly much longer.

### Isolation by distance

**Figure 4:**
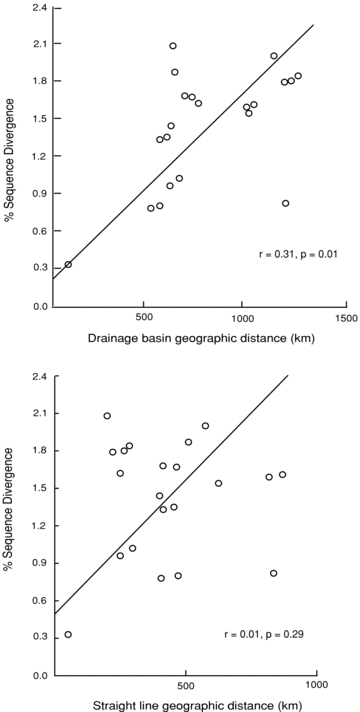
Reduced major axis regressions showing the relationships between percent sequence divergence and two measures of geographic distances among all seven sampling locations. Upper graph: Geographic distances between locations following river drainages. Lower graph: Straight-line geographic distances between locations.

Mantel tests for both genetic distance measures returned highly consistent results, so we present only the results from the corrected “*p*” distances. These tests demonstrated a strong and statistically significant correlation between genetic distances and geographic distance along drainage basins (*r* = 0.652, *P* = 0.013, Fig. 4); however, there was no correlation between genetic distances and straight-line distances between locations (*r* = 0.098, *P* = 0.290, Fig. 4).

## Discussion

This study revealed strong population subdivision for *E. subtaeniata*, throughout most of its range in Indochina, with each sampled location characterized by a unique suite of haplotypes, but with different levels of genetic diversity. The haplotype network indicated that there were many unobserved intermediate haplotypes connecting the 18 haplotypes sampled in the seven locations. It is possible that these “missing” haplotypes still exist and would be uncovered with further sampling of additional locations. However, given the ephemeral nature of suitable freshwater habitats in this region during the Pleistocene (see below) and the strong geographical structuring of haplotypes into local populations (Figs. 2 and 3), it is more likely that these “missing” haplotypes have been lost as the populations in which they occurred went locally extinct. Phylogeographic patterns largely conformed to the geographic proximity of sampled locations and there was a strong relationship between genetic distance and geographic distances along suitable aquatic habitats, suggesting that the geographical configuration of river drainages has significantly influenced the distribution of genetic diversity in this region. We discuss these factors in turn.

### Lower Mekong and Chao Phraya

Maternal lineages of *E. subtaeniata* from the Chao Phraya were genetically more similar to the Lower Mekong than the Lower and Middle Mekong River populations were to each other (Figs. 2 and 3). Although this result is novel compared with some previous studies (e.g., Hurwood et al. 2008), it is not difficult to reconcile with the historical biogeography of the region (Taki 1975; Rainboth 1996; Woodruff 2010). The Quaternary was characterized by extreme glacial-interglacial sea-level fluctuations, with sea levels repeatedly dropping to more than 50 m below present levels for extended periods (Voris 2000). During these periods huge expanses of the Sunda shelf emerged and were traversed by extensive paleo-river drainage systems (Voris 2000; Sathiamurthy and Voris 2006). Woodruff (2010) stresses that an emergent Sundaland has been the predominant Quaternary condition for this region, while the present-day geography has been typical of only 2% of the past million years. As such, the Pleistocene was characterized by extensive lowland connections between what are now the relatively isolated Chao Phraya and Lower Mekong basins. Percent sequence divergence between the Chao Phraya and two Lower Mekong locations (Table 2) suggest that maternal lineages in the Chao Phraya and Lower Mekong diverged between 350,000 and 700,000 years ago, consistent with the timing of the repeated sea-level fluctuations that characterized the last million years of the Pleistocene. Dodson et al. (1995) and McConnell (2004) documented genetic evidence of similar Pleistocene connections via paleo-drainages on the Sunda Shelf for freshwater fish. In addition, faunal similarities between the Chao Phraya and Lower Mekong (Taki 1975; Zakaria–Ismail 1994; Rainboth 1996) support this connection.

### Middle Mekong

Haplotypes from the Middle Mekong and its Chi and Mun tributaries (Fig. 1) also clustered together but did not form a strongly supported monophyletic phylogroup (Fig. 2). Genetic distances among locations (1.0–1.5%) indicated that they have been isolated for at least 500,000 years but possibly longer. The only exception was the close relationship between the geographically proximate Ban Mai and Ban Nong Pueng on the Chi tributary (Figs. 1–3). Interestingly, these two locations also had among the highest haplotype and nucleotide diversities (Table 1), suggesting either larger population sizes or that these populations are older than others sampled in our study. Although the single haplotype (nine) sampled from Ban Badan on the Mun tributary clustered with other Middle Mekong samples in the rooted MP tree (Fig. 2), MP bootstrap support was low (77%) and this haplotype connected the remaining Middle Mekong haplotypes to those from the Lower Mekong and Chao Phraya in the SP network (Fig. 3), and the ML tree (not shown). Previous studies have reported conflicting results regarding the affiliations between the Khorat Basin Middle Mekong and its Mun tributary, with cyprinid fish populations being variously closely related (Adamson et al. 2009) or highly divergent (Hurwood et al. 2008). The Khorat Basin has had a dynamic history and the highly eroded flat terrain seen today is not representative of the Quaternary, when it was strongly dissected by river valleys and mountain ranges (Fontaine and Workman 1978; Hutchison 1989). These features provided numerous opportunities for the isolation of riverine and semiaquatic taxa but did not necessarily result in congruent phylogeographic structures among species.

### Middle Mekong versus lower Mekong populations

Haplotypes from the Lower Mekong locations also clustered together; however, they did not cluster with the Middle Mekong locations (Figs. 2 and 3). The Middle and Lower Mekong basins are currently separated by the Khone Falls, a 10-km stretch of extensive rapids with a width of up to 14 km and a drop in elevation of >20 m that formed as the result of two broad periods of volcanism: an older cycle from 2 to 1 million years ago and a more recent cycle from 700,000 to 600,000 years ago (Fontaine and Workman 1978, p. 580) with some isolated events as recent as 5,000 years ago. Sequence divergences among the Lower and Middle Mekong populations ranged from 1.6 to 2.2%, indicating that populations diverged at least 1 million years ago but possibly much longer. These results suggest that the lava flows that formed the Khone Falls created a partial barrier to dispersal for *E. subtaeniata*. Previous studies have also reported strong genetic discontinuities between Lower and Middle Mekong populations for freshwater fish (Adamson et al. 2009), although this result is not universal (Hurwood et al. 2008), suggesting that the Khone Falls presents a complete or partial barrier for many but not all freshwater species. *Enhydris* species are largely restricted to wetlands and in the areas away from the Mekong flood plain wetlands are patchy and much of the terrain has substantial relief. Thus, the Mekong river flood plain may represent an old and previously continuous wetland connection between the Middle and Lower Mekong.

### Limitations of the dataset

As in most studies, sampling at additional localities (for example, in this study directly below Khone Falls) would likely prove interesting and perhaps further clarify barriers to gene flow. However, the collection sites for *Enhydris* species are to a large extent a function of where the snakes can be found and legally collected. The distribution of wetland habitats and these semiaquatic snakes is not continuous but rather patchy. Thus, the distribution of distances between collections sites is uneven and closely dependent on topography. In addition, adding one or more nuclear genes to the dataset would lend the results more robust. However, it is unlikely that doing so would change the overall results or conclusions.

## Conclusions

The substantial genetic differences among the populations of *E. subtaeniata* in Indochina are perhaps best understood in the context of the Quaternary terrain associated with the Upper and Lower Mekong, the Chao Phraya River, and the Sunda Shelf. The landscapes of both the Khorat Basin of the Upper Mekong and the Great Lake Basin (Tonle Sap) of the Lower Mekong consisted of strongly dissected river valleys separated by numerous low mountain ranges in the early and mid Quaternary (Fontaine and Workman 1978; Hutchison 1989). This highly divided terrain, with substantial topographic relief and numerous distinct catchments certainly provided an abundant opportunity for the isolation of riverine and semiaquatic taxa. During most of this period much of the Sunda Shelf consisted of lowland river valleys that served to connect freshwater faunas associated with the tributaries of the Siam River, including the Mekong and Chao Phraya Rivers. It is thus likely that the contemporary genetic patterns that we observed between *E. subtaeniata* populations in Indochina is primarily a product of their histories in a terrain that imposed abundant opportunities for genetic isolation and independent evolution, as well as lowland connections across now drowned river valleys.

## Appendix A1

Specimen locality data are provided below. The names in bold type are the seven names used on the map in Figure 1. Museum specimen numbers are also provided. Museum acronyms are: FMNH, Field Museum of Natural History and THNHM, Thailand Natural History Museum.

Thailand: Central Basin, Nakon Sawan provience, **Bung Boraphet**; Bang Klong Khut (15°41′49.5″N 101°19′83.3″E), THNHM 12274–12316.

Khorat Basin north, Khon Kaen area, Nong Bua Lam Phu provience, **Ban Mai** (17°10′01″N 102°24′39″E) THNHM 262429–430, 262448–454. Khon Kaen provience, **Ban Nong Pueng** (nr. 16°48′32″N 102°28′ 09″E), THNHM 262460–469.

Khorat Basin south, Nakon Ratchisma provience, **Ban Badan** (14°31′04″N 101° 58′25″E), THNHM 262425–426, 263743–745, 263747–749.

Cambodia: Siem Reap provience, Siem Reap, **Tonle Sap** (13°15′00″N 103°50′00″E), FMNH 259245–246.

Laos: Champasak provience, Conservation area nr **Dong Khanthung** (14°07′00″N 105°29′00″E), FMNH 255037.

Vietnam: Kien Giang, nr. An Minh, **U. Minh Thong** Nature Reserve (09°38′52″N 105°08′35″E), FMNH 259082–087.

Habitat information for the major collecting sites follows:

***Bung Boraphet, Thailand***.–The Bung Boraphet wetland (5 collecting nights) is the largest freshwater wetland system in Thailand; it is internationally recognized for its diverse flora and fauna (Sriwongsitanon et al. 2007). Bung Boraphet is located near the junction of the Ping, Nam and Yom Rivers in the Central Plain, Chao Phraya River Basin, of Thailand (Fig. 1). Today, the center of the wetland is a large, shallow lake (mean depth of 2.0 m) with several islands bordered by rice paddies, marsh areas, and grassland. The wetland is approximately 20 km in length and 7 km at its widest point, depending on flood conditions. Historically the area was a natural wetland that annually flooded during the wet season and then became a grassy plain with scattered ponds and wetlands during the dry season. The current lake was created by a dam built in 1926–30 and further modified later by water level control structures and road construction (Sriwongsitanon et al. 2007).

***Khon Kaen area (Khorat Basin, Thailand)***.— The Khon Kaen area is located in the drainage basin of the Chi River, one of the two primary rivers in the Khorat Basin (Fig. 1); the Chi joins with the Mun river in the southeastern corner of the Khorat Basin and then joins the mainstream of the Mekong River (Fig. 1). We collected snakes at Ban Mai and Ban Nong Pueng located around the perimeter of Ubon Ratana Dam Reservoir (410 km^2^) to the northwest of Khon Kaen.

***Ban Badan, (Khorat Basin, Thailand)***.— The Ban Badan area (6 collecting days in 2003; 20 collecting days in 2004) is in the Mun River Basin (Fig. 1). The area in and around Ban Badan village is flat, agricultural country with a small reservoir, ditches, ponds, rice paddies, and streams. Collecting localities included a small reservoir, three localities in rice paddies and adjacent ditches, and two in small streams, all located within a several km radius of Ban Badan village.

***Sites in Laos, Cambodia, and Vietnam***.—Bryan Stuart (Stuart *et al*., 2000; 2004;) collected a series of *E. subtaeniata* from Dong Khanthung, Laos. This site is at the lower edge of the Middle Mekong Basin, west of the Mekong River, downstream from where the Mun and Chi Rivers join the Mekong, but just above the Khone Falls (Fig. 1). Stuart also obtained snakes from the Lower Mekong Basin at Tonle Sap, an extensive lake/wetland system in Cambodia connected to the Mekong River (Stuart, 2000; Brooks et al., 2007) and from U. Minh Thong National Park, Viet Nam, in the Mekong River delta.

## Appendix A2

Primers used to generate PCR products and mtDNA sequences. Internal primers used for sequencing only are indicated with an asterix (*)

**Table:**

Locus	Primer	Sequence: 5′ > 3′	Source
ATPase	LYS2F	TAGCCTTTTAAGTTGAAGA	Alfaro et al. (2004)
	CO31R	GTGGAGTTGGTGGGTCATTA	Alfaro et al. (2004)
	L-ATPint*	CTACAGGACAAAAATGATCCA	(this study)
	H-ATPint*	CTAGGGCTATATTTATTGATAGTTG	(this study)
ND4	ND4I	TGACTACCAAAAGCTCATGTAGAAGC	Forstner et al. (1995)
	tRNA-Leu	TACTTTTACTTGGATTTGCACCA	Forstner et al. (1995)
Cytochrome *b*	L14910	GACCTGTGATMTGAAAAACCAYCGTTGT	Burbrink et al. (2000)
	H16064	CTTTGGTTTACAAGAACAATGCTTTA	Burbrink et al. (2000)
	L15584*	TCCCATTYCACCCATACCA	Burbrink et al. (2000)
	H15149*	CCCTCAGAATGATATTTGTCCTCA	Kocher et al. (1989)

## Appendix A3

GenBank accession numbers for *Enhydris subtaeniata* haplotypes generated in this study.

**Table:**

Haplytype	ATPase6	ND4	Cyt*b*
Esub_Haplotype 1	JN191578	JN400721	JN392073
Esub_Haplotype 2	JN191579	JN400722	JN392074
Esub_Haplotype 3	JN191580	JN400723	JN392075
Esub_Haplotype 4	JN191581	—	JN392076
Esub_Haplotype 5	JN191582	JN400724	JN392077
Esub_Haplotype 6	JN191583	JN400725	JN392078
Esub_Haplotype 7	JN191584	JN400726	JN392079
Esub_Haplotype 8	JN191585	JN400727	JN392080
Esub_Haplotype 9	JN191586	JN400728	JN392081
Esub_Haplotype 10	JN191587	JN400729	JN392082
Esub_Haplotype 11	JN191588	—	JN392083*
Esub_Haplotype 12	JN191589	JN400730	JN392083*
Esub_Haplotype 13	JN191590	JN400731	JN392084
Esub_Haplotype 14	JN191591	JN400732	JN392085
Esub_Haplotype 15	JN191592	JN400733	JN392083*
Esub_Haplotype 16	JN191593	JN400734	JN392086
Esub_Haplotype 17	JN191594	JN400735	JN392087
Esub_Haplotype 18	JN191595	JN400736	JN392088

* Note that cytochrome *b* haplotypes 11, 12 and 15 are identical.
